# Evidence of avian and human influenza A virus infection in farmed Siamese crocodiles (*Crocodylus siamensis*) in Thailand

**DOI:** 10.1371/journal.pone.0317035

**Published:** 2025-01-07

**Authors:** Metawee Thongdee, Somjit Chaiwattanarungruengpaisan, Natthaphat Ketchim, Nareerat Sangkachai, Nlin Arya, Wanna Sirimanapong, Witthawat Wiriyarat, Pilaipan Puthavathana, Weena Paungpin

**Affiliations:** 1 Faculty of Veterinary Science, The Monitoring and Surveillance Center for Zoonotic Diseases in Wildlife and Exotic Animals, Mahidol University, Nakhon Pathom, Thailand; 2 Faculty of Veterinary Science, Department of Pre-clinic and Applied Animal Science, Mahidol University, Nakhon Pathom, Thailand; 3 Faculty of Veterinary Science, Department of Clinical Sciences and Public Health, The Veterinary Aquatic Animal Research Health Care Unit, Mahidol University, Nakhon Pathom, Thailand; 4 Faculty of Medical Technology, Center for Research and Innovation, Mahidol University, Nakhon Pathom, Thailand; University of South Dakota, UNITED STATES OF AMERICA

## Abstract

Crocodilians are susceptible to a range of virus infection including influenza A virus (IAV). However, little is known about the ecology and epidemiology of IAV in crocodile species. This study aimed to investigate IAV infection in farmed Siamese crocodiles in central Thailand. We collected plasma samples and pharyngeal swab samples from Siamese crocodiles residing in 13 crocodile farms in 9 provinces of central Thailand during 2019. Additional archival plasma samples of Siamese crocodiles collected in 2012 and 2018 were also included in the study. Plasma samples were screened for influenza A antibodies by a hemagglutination inhibition (HI) assay and positive were evaluated by a cytopathic effect/hemagglutination based-microneutralization (MN) assay. Swab samples were tested for influenza viral RNA by a real-time RT-PCR assay targeting the influenza matrix (M) gene. Among 246 tested plasma samples, the overall seroprevalence of antibodies against IAV in farmed Siamese crocodiles was 17.5% (43/246). The most common hemagglutinin (HA) subtype was H2 (46.5%, 20/43) followed by H9 (39.5%, 17/43), human H1 (14%, 6/43) and H1 (7%, 3/43). Multiple HA subtypes were also detected in 7% (3/43) of infected crocodiles with combination of H1 and H2 subtypes. All 126 tested swab samples were negative for influenza viral RNA. In addition, we demonstrated the ability of wild-type IAV subtypes (H1, H2, H9 and human H1) to infect primary Siamese crocodile fibroblast cells. To our knowledge, this is the first report of serological evidences of avian and human IAV infection in Siamese crocodiles. Our findings highlighted the role of crocodile species in the ecology of IAV particularly the potential to serve as the reservoir or mixing vessel for the viruses that significantly threaten both human and animal health.

## Introduction

Influenza A virus (IAV) has the ability to infect a wide range of host species, including avian, human, and mammalian populations [1]. IAV has been well documented in avian species and mammals. However, new influenza viruses and host species are still being identified. The latest discovery of the genome of IAV-like H17 in three little yellow-shouldered bats (*Sturnia lilium*) in Guatemala and IAV-like H18 in one flat-faced bat (*Artibeus planirostris*) in Peru suggested that bats may constitute another reservoir of unknown influenza viruses with greater genetic diversity [2,3]. Wild aquatic birds are the primary natural reservoir for IAV subtypes [4], but not for the two new subtypes (H17N10 and H18N11) found in bats [2,3].

The continuous circulation of influenza viruses among animal hosts could lead to the emergence of new strains with epidemic and pandemic potential due to periodic genetic changes such as evolution, adaptation, and gene reassortment [5]. The previous pandemics in human and numerous outbreaks of influenza in domestic and wild animals have been linked to inter- and intra-species transmission of avian influenza viruses [1,6]. Hence, virus subtypes with high zoonotic potential are public health concern, including highly pathogenic avian influenza (HPAI) H5 and H7 and low pathogenic avian influenza (LPAI) H1, H2, H3 and H9 [7–10]. Furthermore, information on virus subtypes identified in new hosts or new geographical areas or endemic areas will be valuable for tracing the evolution of influenza viruses in animals as well as discovering the new genetic traits of known circulating viruses [7]. In addition, the effect of seasonal variation on the transmission of influenza virus should be also considered. Climatic and ecological factors such as temperature, humidity, and precipitation influence the transmission dynamics of the disease [11,12]. The environmental factors drive the virus transmission through several mechanisms including contact rates between susceptible and infected hosts, virus survival and host immunity [13]. Understanding of the potential environmental factors as well as mediating mechanisms should be useful to forecast disease dynamics in different regional settings. Thus, surveillance of IAV infection in many animal species, especially the animals that share their habitat with humans, domestic avian species and wild birds, is still needed.

Little is known about IAV in reptilia which crocodilians (alligators, caimans, crocodiles, and gharials) are one of the class members. Crocodilians have slow rate of genome evolution and share several physical characteristics with birds such as egg structure, embryonic development and similar antibody isotypes [14]. Thus, crocodilians are the closest living relatives of birds [15,16]. Many crocodilian species share the same aquatic ecosystems with the wild birds, which are the reservoir for numerous subtypes of IAV. Moreover, birds often serve as an important food source for many crocodilians [17]. Based on the information of evolution, biology and ecology of crocodilians, these animal species are logically considered to be susceptible to influenza virus infection and potentially provide virus linkage between birds and mammals.

A few studies have demonstrated the infection of IAV among crocodilian species. The previous study by Davis LM and Spackman E in 2008 revealed the detection of influenza viral RNA from blood and serum samples of four captive crocodilians (*Alligator sinensis*, *Caiman latirostris*, *Crocodylus niloticus* and *Paleosuchus trigonatus*) in Florida [18]. The other study by Temple BL, et al (2015) demonstrated the susceptibility of American alligator (*Alligator mississippiensis*) to IAV infection in ovo by embryonated alligator eggs and in vitro by alligator primary embryonic fibroblasts [14]. Moreover, this study also provided the evidence of replication of IAV in alligator primary embryonic fibroblasts, confirming that alligator cells can support the complete infectious lifecycle of influenza virus [14].

Thailand is one of the biggest crocodile farming sectors in the world which several crocodile farms in Thailand are well-known tourist attractions. By December 2023, Department of Fisheries reported the presence of over 1.2 million Siamese crocodiles (*Crocodylus siamensis)* across 1,415 registered farms in Thailand [19]. Majority of crocodile population have been raised for the purpose to supply raw materials for the leather industry. However, demand for other crocodile products such as meat and blood have also been growing to supply for alternative dietary and medication [20]. Most of farmed crocodiles live in open-air semiaquatic environment where the farm area includes both aquatic and terrestrial parts. Farmed crocodiles are normally fed a diet of raw meat which chicken is often used since it is readily available. According to environmental habitat and feeding behavior, IAV infection among farmed crocodiles can be possibly occurred through the ingestion of infected tissues, inhalation of infectious particles or exposure to bird excrement [14,21]. Moreover, increasing of human-crocodile interaction can be the possible source of virus transmission.

In this study, we attempted to detect both antigen and antibody to IAV in farmed Siamese crocodiles; and we also used primary crocodile fibroblast cells to assess the susceptibility of Siamese crocodiles to IAV infection. This study will contribute the knowledge on ecology and epidemiology of IAV in crocodiles. Data obtained from the study will consolidate the existing data on the evidence of influenza viruses in crocodilian species. The prevalence of influenza viruses in crocodiles may provide a clue for which viruses are currently circulating in the populations and environment. This information points out public health implication of influenza transmission through crocodiles and emphasizes the need for increasing active surveillance efforts in order to target more influenza virus subtypes and relevant animal species.

## Materials and methods

### Ethical issues

The use of animal samples for this study was approved by the Faculty of Veterinary Science, Mahidol University Institute of Animal Care and Use Committee (FVS-MU-IACUC); animal Ethics No. MUVS-2019-12-56 and MUVS-2023-12-80 for the use of crocodile samples and MUVS-2021-07-29 for the use of primary crocodile fibroblasts. The study protocol involving influenza virus was approved by the Institutional Biosafety Committee of Mahidol University (IBC#2021–004 and IBC#2023–031).

### The study population and sample collection

Sample collection was carried out from 274 Siamese crocodiles (*Crocodylus siamensis*) residing in crocodile farms located in 9 provinces of central Thailand. Among the study population of crocodiles, 175 crocodiles belonging to 13 crocodile farms were blood and swab sampled during disease surveillance for Chlamydia and other crocodile diseases in 2019. Additional archrival plasma samples derived from 69 and 30 crocodiles from two different farms collected during 2012 and 2018, respectively were included for the study. Demographic data on place and age of individual animal was recorded (Table 1). Sex was not determined. Blood was drawn via caudal vein with syringes containing heparin as anticoagulant. Pharyngeal swab was collected from each animal using sterile polyester tipped swab. Swabs were placed into individual tubes of 1 ml viral transport media (VTM). All samples were kept on ice during transportation to the laboratory within the same day.

**Table 1:** Demographic characteristics of the samples derived from farmed Siamese crocodiles (*Crocodylus siamensis*).

No	Place	Province	Date of collection	Age	Number of samples	
					Pharyngeal swab	Plasma
1	Farm A	Nakhon Pathom	Jan 12, 2012	5 Y	N/A	69
2	Farm B	Nakhon Pathom	Aug 2, 2018	1 Y	N/A	30
3	Farm C	Suphanburi	Jul 19, 2019	1 Y	0	14
4	Farm D	Uthai Thani	Jul 20, 2019	1 Y	13	14
5	Farm E	Sing Buri	Jul 30, 2019	1 Y	0	13
6	Farm F	Saraburi	Jul 30, 2019	2 mo	10	7
7	Farm G	Suphan Buri	Jul 31, 2019	7 mo	14	5
8	Farm H	Saraburi	Sep 4, 2019	4 mo	14	14
9	Farm I	Lopburi	Sep 17, 2019	2 Y	0	14
10	Farm J	Lopburi	Sep 17, 2019	1 Y	14	14
11	Farm K	Nakhon Sawan	Sep 18, 2019	2 Y	8	8
12	Farm L	Nakhon Sawan	Sep 18, 2019	3 mo	15	9
13	Farm M	Chai Nat	Sep 30, 2019	1 Y	7	14
14	Farm N	Ayutthaya	Nov 11, 2019	7 mo	15	14
15	Farm O	Nakhon Pathom	Dec 6, 2019	4 mo	16	7
**Total**					**126**	**246**

N/A = not available, Y = Year, mo = month.

### The study virus

To identify the presence of antibodies to IAV, we selected the low pathogenic avian influenza (LPAI) virus subtypes; H1-H5, H7, H9-H12 and the human H1 influenza virus as the test viruses. All selected virus subtypes were found to circulate among avian and mammal species in Thailand [22–28]. All LPAI viruses were propagated in the allantoic cavity of 9–11 day-old embryonated chicken eggs. The human H1N1 influenza viruses were propagated in MDCK (ATCC CCL-34) cells containing Eagle’s minimum essential medium (EMEM) supplemented with 2 μg/ml of trypsin-TPCK for maintenance of the viral growth. The strains of tested viruses are listed in Table 2. All LPAI viruses were obtained from St. Jude Children’s Research Hospital, TN, USA. The human H1 influenza viruses were obtained from Center for Research and Innovation, Faculty of Medical Technology, Mahidol University.

**Table 2:** List of influenza A viruses used in the study.

Strain	Subtype
A/Aquatic bird/Hong Kong/DI25/2002	H1N1
A/Wild Duck/Shan Tou/992/2000	H2N8
A/Duck/Shan Tou/1283/2001	H3N8
A/Duck/Shan Tou/461/2000	H4N9
A/Duck/Jiangxi/6151/2003	H5N3
A/Ostrich/Zimbabwe/222/1996	H7N1
A/Chicken/Hong Kong/G9/1997	H9N2
A/Duck/Shan Tou/1796/2001	H10N8
A/Duck/Shan Tou/1411/2000	H11N2
A/Red-necked stint/Australia/5745/1981	H12N9
A/Thailand/104/2009	H1N1
A/Thailand/NP-045/2018	H1N1

### Real-time reverse transcription-PCR

A total of 126 pharyngeal swab samples were subjected to viral RNA extraction. Influenza RNA was extracted using the QIAamp viral RNA Mini Kit (QIAGEN), according to the manufacturer’s instructions. Real-time RT-PCR targeting the conserved region of influenza matrix gene was performed using the AgPath-ID™ One-Step RT-PCR Kit (ThermoFisher Scientific) and using the QuantStudio 3 Real-Time PCR System (QuantStudio). The cycle threshold (Ct) value ≤ 38 was considered as a positive result.

### Hemagglutination inhibition (HI) assay

Plasma samples were screened for antibodies to specific HA subtypes (H1-H5, H7, H9-H12 and human H1 subtypes) using HI assay as described [29,30]. Individual plasma samples were pre-treated with the receptor-destroying enzyme (RDE; Denka Seiken, Tokyo, Japan) at a dilution of 1:4 and then incubated at 37°C overnight to remove nonspecific inhibitors. Plasma samples were further incubated at 56°C for 30 minutes to inactivate the process, which was followed by adding phosphate-buffered saline to achieve a 1:20 dilution. Two-fold serial dilution (25 μL) of RDE-treated plasma samples were prepared in a 96-well V-bottom plate starting at a 1:20 dilution and subsequently incubated with 25 μL of the tested virus (4 HA units per 25 μL) at room temperature for 30 min. The hemagglutination titer was read after adding a 0.5% suspension of goose red blood cells and was incubated at 4°C for 1 h. Nonspecific HA activity were tested by mixing RDE-treated plasma samples with 0.5% suspension goose red blood cells. The highest reciprocal of the serum dilution that completely inhibited hemagglutination was recorded as the endpoint titer. Geometric mean titers (GMTs) of each virus subtype were calculated. The HI titers < 20 were assigned as 10. The plasma samples with HI titers ≥20 was further determined for neutralizing antibodies using a microneutralization (MN) assay.

### Microneutralization (MN) assay

All HI-positive plasma samples were tested by MN assay as described [30,31]. The treated plasma samples were twofold serially diluted in duplicate and incubated with the test virus at final concentration of 100TCID50/well for 2 hours at 37°C. After incubation, the plasma-virus mixture was then added to the MDCK cells that were seeded in 96-well plates 24 hours before infection. The cells were incubated with the mixture at 37°C under 5% CO_2_ for 3 days. The appearance of cytopathic effect (CPE) was examined with light microscopy; while the supernatants were collected to test for non-neutralized viruses by hemagglutination assay. The neutralizing titer (NT) was determined as the reciprocal of the highest plasma dilution that achieved ≥50% virus neutralization. The plasma samples with the HI and NT titers ≥20 was considered as seropositive indicating the previous IAV infection.

### Western blot (WB)

The presence of IgY antibody in the plasma samples of the infected crocodiles was confirmed by WB. Antibody protein was evaluated on a discontinuous SDS-PAGE gel in the absence (non-reducing) or presence (reducing) of β-mercaptoethanol. Crocodile plasma was serially 2-fold diluted from the starting dilution of 1:50 to 1:200. The diluted plasma was mixed with 4× non-reducing sample buffer and loaded in electrophoresing gel (8% SDS-PAGE) under the non-reducing condition; while crocodile plasma was mixed with 4× reducing sample buffer and boiled at 98°C for 10 minutes prior to electrophoresing in 12% SDS-PAGE under the reducing condition. The electrophoresed proteins in gel were then blotted onto a nitrocellulose membrane (Bio-Rad, Hercules, CA) using TE77X semi-dry transfer unit (Hoefer Inc). The blotted membrane was blocked with 1% bovine serum albumin in PBS plus 0.1% Tween-20, followed by overnight incubation at 4°C with horseradish peroxidase-conjugated chicken IgY (cat.no. AB6877, Abcam Cambridge, UK) as the detector. The solution mixture containing 3,3’-diaminobenzidine (Sigma-Aldrich), 8% NiCl2 and 30% H2O2 was used as the chromogenic substrate. Chicken serum was included as the control to verify the molecular weight (MW) of IgY molecule.

### Immunofluorescence staining

Crocodile fibroblast cells were trypsinized using 0.05% Trypsin-EDTA and then plated at 2.5 x 10^5^ cells/ml in EMEM medium with antibiotics (100 IU/ml penicillin G, 100 μg/ml streptomycin, 6 μg/ml amphotericin B) in 24-well plates. After 24 hours, crocodile fibroblast cells were inoculated with 100 μl of IAV strain (A/Aquatic bird/Hong Kong/DI25/2002 (H1N1), A/Wild Duck/Shan Tou/992/2000 (H2N8), A/Chicken/Hong Kong/G9/1997 (H9N2), and A/Thailand/104/2009 (human H1N1)) diluted in viral growth media (EMEM supplemented with 2 *μ*g/ml TPCK-trypsin) at a multiplicity of infection [MOI] of 0.05 and cells were incubated at 37°C under 5% CO_2_ condition for 2 hours. Cells were fixed with ice-cold methanol-acetone (1:1) at room temperature for 20 minutes and the fixed cells were washed three times with PBS. To visualize IAV infection, cells in each well were incubated with 1:200 dilution of a mouse anti-nucleoprotein (NP) monoclonal antibody (cat.no. MAB8257, Merck Millipore, Merck KGaA, Darmstadt, Germany) and 1:500 dilution of Alexa Fluor 488-conjugated polyclonal goat anti-mouse IgG (H+L) (cat.no. AB150113, Abcam Cambridge, UK). Nuclear DNA of fixed cells was stained with 1 mg/ml of 4’,6-Diamidino-2-phenylindole (DAPI) (Molecular Probes, OR, USA). The cells were visualized using a Zeiss Axiovert 40 CFL fluorescent light microscope (Carl Zeiss Microscopy, Thornwood, NY).

## Results

### HI and MN assays for antibody against influenza A virus

A total of 246 crocodile plasma including 69 samples (28.0%) collected in 2012, 30 samples (12.2%) collected in 2018 and 147 samples (59.8%) collected in 2019 were tested for IAV antibodies. Overall, 17.5% (43/246) of farmed crocodiles plasma samples tested positive for the presence of IAV antibodies. The seroprevalence was 21.7% (15/69) of the samples from 2012, 26.7% (8/30) of the samples from 2018 and 13.6% (20/147) of the samples from 2019. HA-specific antibodies were detected against avian influenza virus (AIV) H1 (1.2%, 3/246), AIV H2 (8.1%, 20/246), AIV H9 (6.9%, 17/246), and human H1 (TH104) (2.4%, 6/246) (Tables 3 and S1). Among the 43 positive plasma samples, 3 samples (7%) tested antibody positive for more than one HA subtype, with combination of avian H1 and H2. The HI and NT titers of the seropositive samples were in the range of 20–80. The GMT (95% CI) of HI titers against each virus subtype were determined as AIV H1 = 10.08 (9.99–10.18), AIV H2 = 10.73 (10.40–11.08), AIV H9 = 10.70 (10.33–11.08), and human H1 (TH104) = 10.26 (10.03–10.49). Similarly, the GMT (95% CI) of NT titers against each virus subtype were also determined as AIV H1 = 20.00 (20.00–20.00), AIV H2 = 36.05 (29.00–44.81), AIV H9 = 25.54 (21.43–30.45), and human H1 (TH104) = 50.40 (34.62–73.37). None of the crocodile plasma samples tested positive to IAV HA subtypes including avian H3, H4, H5, H7, H10, H11, and human H1 (NP045).

**Table 3:** The number of crocodiles with seropositive against subtypes of influenza A virus divided by farm.

No	Place	Province	Date of collection	Plasma	Number of positive samples with influenza subtypes**				% positive by farm (Total)
					AIV H1	AIV H2	AIV H9	Human H1	
1	Farm A	Nakhon Pathom	Jan 12, 2012	69	-	-	9	6	21.7
2	Farm B	Nakhon Pathom	Aug 2, 2018	30	-	-	8	-	26.7
3	Farm C	Suphan Buri	Jul 19, 2019	14	-	-	-	-	0
4	Farm D	Uthai Thani	Jul 20, 2019	14	-	-	-	-	0
5	Farm E	Sing Buri	Jul 30, 2019	13	1*	5	-	-	38.5
6	Farm F	Saraburi	Jul 30, 2019	7	-	-	-	-	0
7	Farm G	Suphan Buri	Jul 31, 2019	5	-	2	-	-	40
8	Farm H	Saraburi	Sep 4, 2019	14	2*	6	-	-	42.9
9	Farm I	Lopburi	Sep 17, 2019	14	-	-	-	-	0
10	Farm J	Lopburi	Sep 17, 2019	14	-	1	-	-	7.1
11	Farm K	Nakhon Sawan	Sep 18, 2019	8	-	-	-	-	0
12	Farm L	Nakhon Sawan	Sep 18, 2019	9	-	-	-	-	0
13	Farm M	Chai Nat	Sep 30, 2019	14	-	2	-	-	14.3
14	Farm N	Ayutthaya	Nov 11, 2019	14	-	4	-	-	28.6
15	Farm O	Nakhon Pathom	Dec 6, 2019	7	-	-	-	-	0
				246					(17.5)

AIV = avian influenza virus, Human = human influenza virus.

*: Individual crocodile gave seropositive results for both virus subtypes.

**: Number of individual crocodiles gave seropositive with HI and NT titers of ≥20.

**Fig 1:**
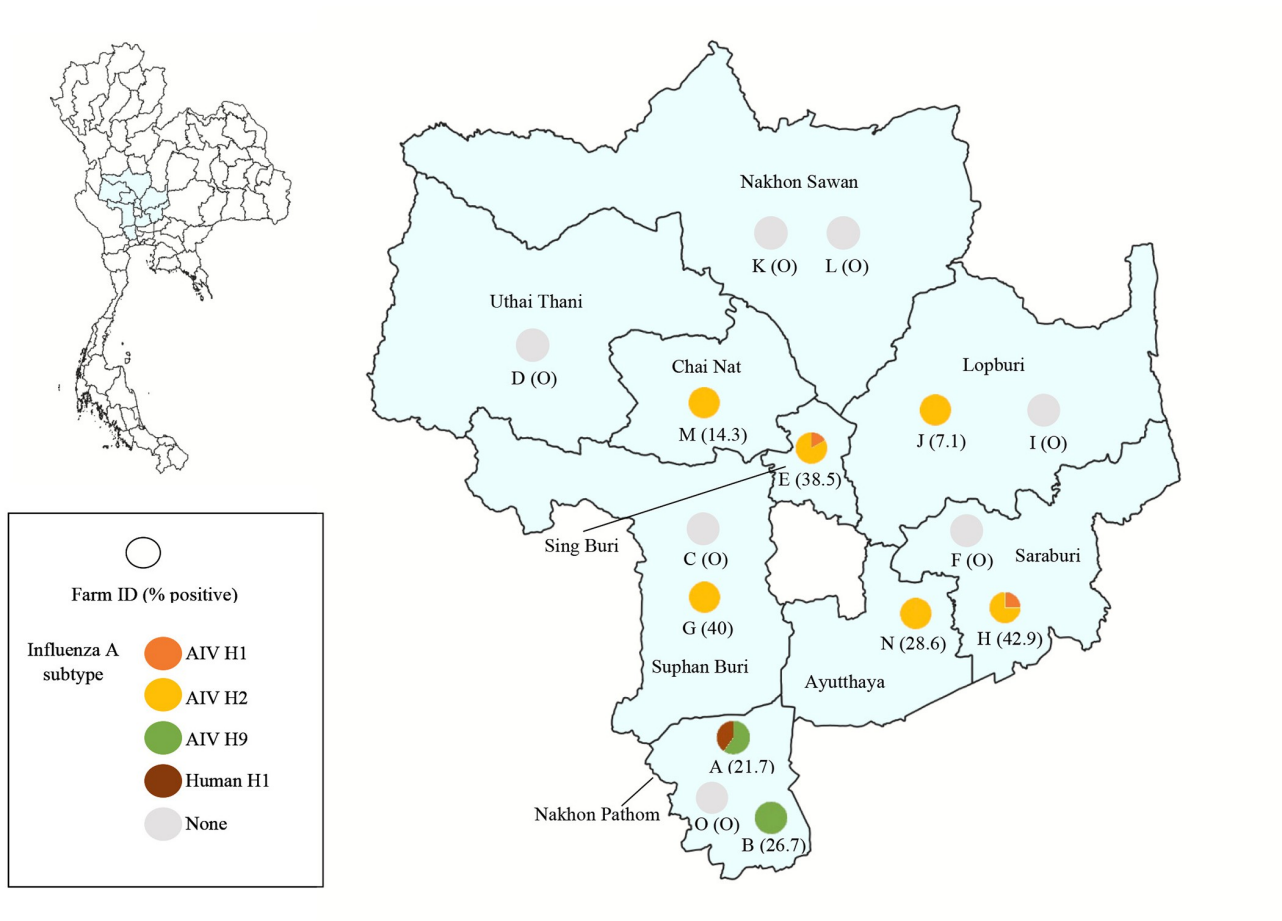
Map of nine provinces of central Thailand and distribution of seropositive cases against influenza A subtypes in plasma of farmed Siamese crocodiles by HI and MN assay. The circle represents the farm location and the positivity observed with the identified influenza A subtypes. The map was generated using QGIS (Quantum Geographic Information System) version 3.34.13 (https://qgis.org). Geographical materials used for creating the map (e.g., shapefiles) were supported by the DIVA-GIS database (https://www.diva-gis.org/). Source of base map: GADM (https://gadm.org/license.html).

Eight out of 15 (53.3%) of crocodile farm sampled were IAV seropositive. The positive farms were located in 7 provinces of central Thailand; Nakhon Pathom (n = 2), Sing Buri (n = 1), Suphan Buri (n = 1), Saraburi (n = 1), Lopburi (n = 1), Chai Nat (n = 1), and Ayutthaya (n = 1) (Fig 1 and Table 3). Of those positive farms, two farms in Nakhon Pathom province were sampled in 2012 and 2018 while the other six farms were sampled in 2019. The seropositivity rate observed among positive farm ranged from 7.1–42.9%. The lowest seropositivity (7.1%, 1/14) was found in the crocodile Farm J in Lopburi province, whereas the highest seropositivity (42.9%, 6/14) was identified in the crocodile Farm H in Saraburi province (Fig 1 and Table 3). Crocodiles sampled at one farm in Uthai Thani province and two farms in Nakhon Sawan province were all seronegative (Fig 1).

AIV H9 was the most common subtype found in crocodiles sampled in 2012 and 2018 with a positive rate of 13.0% (9/69) and 26.7% (8/30), respectively. While AIV H2 was the most common subtype found in crocodiles sampled in 2019 with a positive rate of 13.6% (20/147) (Tables 3 and S1). Additional HA subtypes, human H1 (TH104) (8.7%, 6/69) and AIV H1 (2.0%, 3/147) were identified in crocodiles sampled in 2012 and 2019, respectively. The presence of multiple HA-subtypes was observed only in crocodiles sampled in 2019 with combination of AIV H1 and AIV H2. Antibodies against both the AIV H1 and AIV H2 were detected in one out of 13 samples (7.7%) from Farm E in Sing Buri province and two out of 14 samples (14.3%) from Farm H in Saraburi province (Tables 3 and S1).

### WB assays for crocodile immunoglobulin Y (IgY)

**Fig 2:**
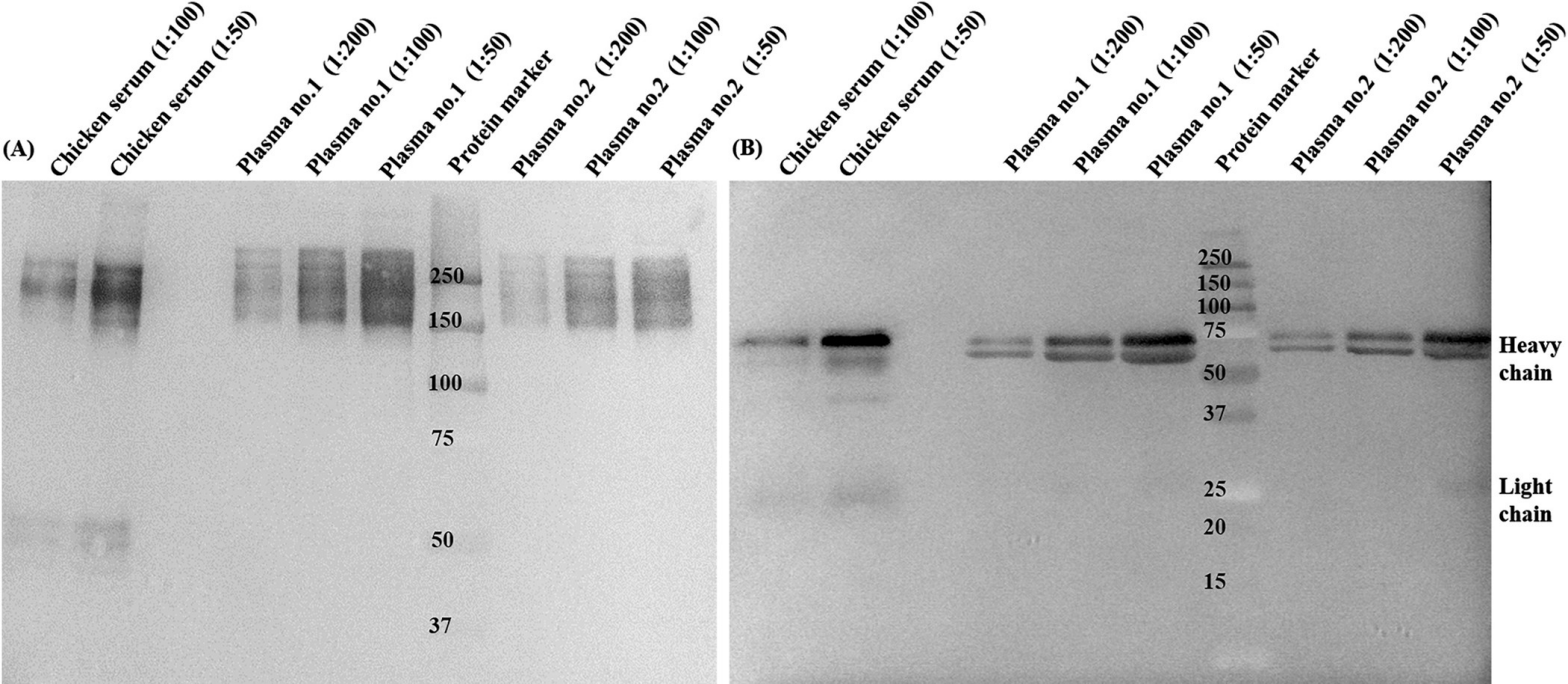
Western blot analysis showing the presence of crocodile IgY antibody in the infected crocodile plasma. Crocodile plasma samples (No. 1 and No. 2) at 1:50, 1:100 and 1:200 dilutions were run on SDS-PAGE gels for protein separation. The blotted membranes were probed with HRP conjugated chicken IgY antibody and protein bands were visualized after adding chromogenic substrate. (A) On non-reducing 8% SDS-PAGE gel, the crocodile IgY molecule (Y) was observed at MW of approximately 180 kDa (B) On reducing 12% SDS-PAGE gel, crocodile IgY heavy chain and light chain were observed at MW of approximately 60–70 kDa and 25 kDa, respectively. Chicken serum samples at 1:50 and 1:100 dilutions were included as control which the chicken IgY were detected with HRP conjugated-goat anti-chicken IgY antibody. A standard protein marker with the indicated MW (in kDa) was included in each SDS-PAGE gel. Raw western blot images are available in S1 Fig.

We confirmed the presence of IgY antibody in the plasma samples of the infected crocodiles by WB. Two plasma samples (No. 1 and No. 2) obtained from individual infected crocodiles were used for protein separation and proteins were detected with HRP conjugated chicken IgY antibody. Each crocodile plasma sample was prepared to the dilution of 1:50, 1:100 and 1:200 while the chicken serum sample was prepared to the dilution of 1:50 and 1:100. The crocodile plasma protein was separated by SDS-PAGE under non-reducing and reducing conditions and the chicken serum protein was analyzed in parallel as a control. Under non-reducing condition, the crocodile plasma produced protein bands of approximately 180 kDa by mobility (Fig 2A). The observed band at 180 kDa likely represent the crocodile IgY molecule [32,33] which showed the similar banding pattern on the gel as the chicken serum control. Under reducing condition, the crocodile plasma produced protein bands of approximately 70, 60 and 25 kDa by mobility which a faint band of 25 kDa was observed only in the 1:50 dilution of crocodile plasma (Fig 2B). The banding pattern of crocodile plasma proteins were similar to the banding pattern of corresponding proteins in chicken serum control (Fig 2B). The observed bands at 70 and 60 kDa likely represent the IgY heavy chain and the band at 25 kDa likely represent the IgY light chain. Based on the SDS-PAGE analysis and immunoblotting, the presence of crocodile IgY antibody could be demonstrated under non-reducing and reducing conditions by using HRP conjugated-chicken IgY antibody as the detector.

### Real-time RT-PCR assay

A total of 126 pharyngeal swab samples collected in 2019 were tested for influenza A viral RNA by real-time RT-PCR assay targeting influenza matrix gene. The swabs were taken from crocodiles in 10 farms in 8 provinces including Farm D in Uthai Thani (10.3%, 13/126), Farm F and H in Saraburi (7.9%, 10/126 and 11.1%, 14/126), Farm G in Suphan Buri (11.1%, 14/126), Farm J in Lopburi (11.1%, 14/126), Farm K and L in Nakhon Sawan (6.3%, 8/126 and 11.9%, 15/126), Farm M in Chai Nat (5.6%, 7/126), Farm N in Ayutthaya (11.9%, 15/126) and Farm O in Nakhon Pathom (12.7%, 16/126). Among 10 crocodile farms sampled, 5 farms (Farm G, H, J, M and N) were IAV seropositive and 5 farms (Farm D, F, K, L and O) were IAV seronegative. Of 126 pharyngeal swab samples, 64 out of 126 (50.8%) were from crocodiles in positive farms and 62 out of 126 (49.2%) were from crocodiles in negative farms. The detection of influenza A viral RNA by real-time RT-PCR showed negative results for all swab samples. This finding suggested that none of the farmed crocodiles were actively infected with influenza A virus at the time of sample collection.

### Immunofluorescence staining

**Fig 3:**
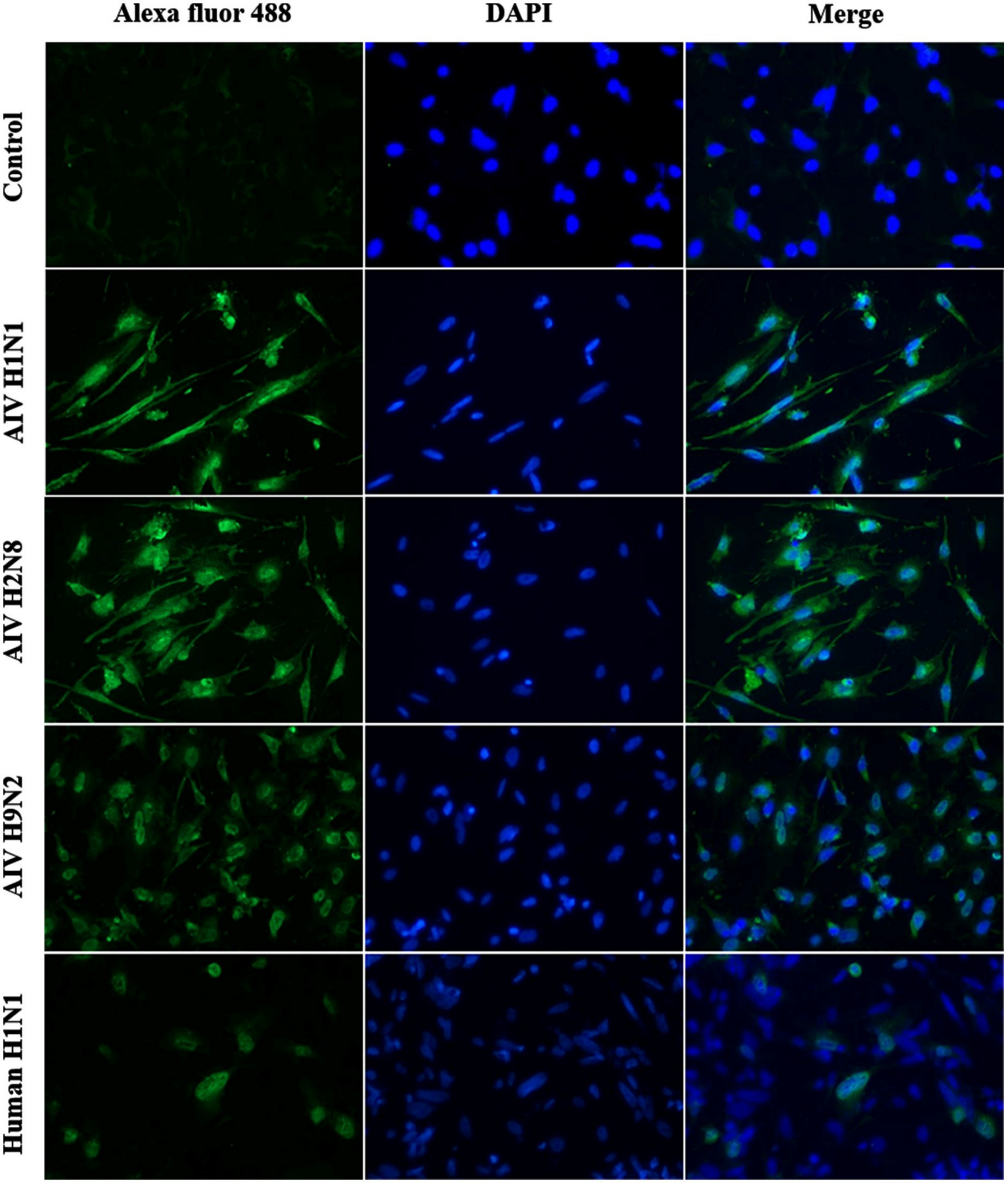
Immunofluorescence staining of primary crocodile fibroblast cells at 24 hours post-infection with AIV H1N1, AIV H2N8, AIV H9N2 and human H1N1 (TH104). Mock-infected cells were included as negative control. The influenza virus nucleoprotein (NP) localization was observed using Alexa fluor 488-conjugated mouse antibody (in green). Cell nucleus DNA was counterstained with DAPI (in blue). Magnification, X200.

Based on the IAV antibodies presented in the plasma samples of the farmed crocodiles, we investigated the ability of avian and human influenza viruses to infect crocodile cells. The primary crocodile fibroblast cells were infected with AIV H1N1, AIV H2N8, AIV H9N2 and human H1N1 (TH104) at MOI 0.05. Infections were observed using immunofluorescence to detect expression of nucleoprotein (NP) of influenza virus in infected cells after 24 hours post-infection. Non-infected primary crocodile fibroblast cells were included as control. The result showed that the NP (green fluorescence) localized in both nucleus and cytoplasm of AIV H1N1 and AIV H2N8 infected crocodile cells whereas the majority of the NP localized in the nucleus of AIV H9N2 and human H1N1 (TH104) infected crocodile cells. No influenza virus NP was observed in non-infected crocodile cells (Fig 3). The number of NP positive cells with human H1N1 (TH104) infection was lower frequency comparing to the number of NP positive cells with avian H1N1, H2N8 and H9N2 infection.

## Discussion

In this study, we report on serological evidence of influenza A viruses in farmed Siamese crocodiles from central Thailand. We identified antibodies to avian H1, H2, H9 and human H1 subtypes in the seropositive plasma samples of the farmed crocodiles. The further analyses on wildtype virus infection in primary Siamese crocodile fibroblast cells confirmed the ability of avian H1, H2, H9 and human H1 influenza viruses to infect and replicate within crocodilian host cells. Our findings indicated that Siamese crocodiles are susceptible to both avian and human influenza virus infection. Moreover, the virus subtypes causing infection in crocodiles are highly concerned for their impact to both animal and public health.

Only one previous study in 2008 reported the IAV infection in crocodilians in USA [18]. The study detected influenza viral RNA in either blood or serum of four individual captive crocodilians including the Chinese alligator (*Alligator sinensis*), smooth-fronted caiman (*Paleosuchus trigonatus*), broad-snouted caiman (*Caiman latirostris*), and Nile crocodile (*Crocodylus niloticus*) (4/37, 10.8%) [18]. However, this study could not isolate the virus from RT-PCR positive samples in embryonated chicken eggs [18]. In addition, this study also detected the antibody against IAV using agar gel immunodiffusion assay which 3 out of 7 serum samples showed non-specific reaction to the tested antigen (*A*. *sinensis*, *P*. *trigonatus* and *C*. *niloticus*) [18].

Although our real-time RT-PCR detection showed negative result from all pharyngeal swab samples, our serological findings provide the evidence of the past exposure to influenza viruses among crocodile population in Thailand. More than 20% of the crocodiles sampled in 2012 and 2018 harbored antibody to influenza viruses which the common virus subtype found in both sampling periods was avian H9 virus. While 9% of the crocodiles sampled particularly in 2012 showed antibody against human H1 virus. Approximately 14% of the crocodiles sampled in 2019 were positive to influenza viruses which the common virus subtype found in this sampling period was avian H2 virus. In addition, 2% of the crocodiles sampled in 2019 harbored antibody to multiple virus subtypes which was the combination of avian H1 and H2 viruses.

The presence of AIV subtypes including H1, H3, H4, H7, H9, H10, H11 and H12 have been identified in domestic avian species and wild birds in Thailand [23–28]. The carcass or tissue derived from infected avian species may present the risk of the virus spreading to humans, animals, and the surrounding environment. Consumption of raw chicken carcasses with contamination of avian influenza viruses could be most likely to cause virus infection in farmed crocodiles. In addition, the fecal-oral route from wild birds may also the possible cause of virus exposure in farmed crocodiles. On the other hand, the evidence of high seroprevalence of avian H2 in farmed crocodiles was consistent with our previous study in free-ranging macaques in Thailand. The earlier finding indicated that macaques in several parts of Thailand had statistically significant differences in the prevalence of antibody against avian H2 subtype compared to other influenza virus subtypes [22]. The circulation of AIV especially H2 and H9 subtypes in varied animal reservoir hosts may cause public concerns since both subtypes are at risk of mutating and reassorting with other subtypes, which could potentially lead to the emergence of pandemic strains [9,10]. Thus, an active influenza surveillance program is needed to identify reservoir hosts of avian H2 and H9 subtypes in domestic avian species and wild birds in Thailand.

For human influenza virus exposure, antibody against human H1 (TH104) was only identified in farmed crocodiles in 2012. Hemagglutinin nucleotide sequence of the strain of A/Thailand/104/2009(H1N1), human H1 (TH104) (accession number GQ169382) was 99.6% identical to that of A/California/7/2009 pandemic virus (accession numbers FJ966974). Currently, the influenza A (H1N1) pdm09 virus continues to circulate worldwide as a seasonal flu virus. The presence of antibody against human H1 virus in the farmed crocodiles during 2012 was in line with that found in the human population at the same period [34,35]. The source of human H1 virus infection in farmed crocodiles likely came from the close contact with infected persons who engaged in activities such as feeding with the farmed crocodiles. However, we have earlier identified human H1 viral infection in domestic and wild animals in Thailand including elephants, captive tiger, dromedary camel, Eld’s deer and macaques which virus exposure likely came from animal caretakers or tourists who engaged in activities involving with those animals [22,36–38]. Although the source of virus infection and the route of virus transmission are under investigated among farmed crocodiles, it should take into account that crocodiles can possibly expose to viruses through sharing habitats/environment with infected humans or animal reservoirs especially domestic avian species and wild birds.

Our western blot result confirmed the presence of immunoglobulin Y (IgY) in plasma samples of the infected crocodiles. It is known that IgY found in amphibians, birds and reptiles is the functional immune response equivalent to mammalian IgG [32]. The detectable antibodies against influenza virus in farmed crocodiles by HI and NT assays suggest that crocodile IgY antibodies could bind the hemagglutinin and neuraminidase of influenza A viruses. Several studies demonstrated that IgY plays a crucial role in neutralization and protection against influenza infection [39–41]. In 2011, Tsukamoto, et al has succeeded to produce IgY neutralizing antibodies against the 2009 H1N1 influenza virus from ostrich eggs immunized with swine influenza vaccine strain [39]. Further *in vitro* testing has demonstrated that the ostrich IgY inhibited the hemaggregations by the swine influenza virus and inhibited cytopathological effects on MDCK cells of infection with pandemic influenza virus A/H1N1 2009 [39]. The other study by Yang, et al (2014) has shown that the IgY antibody obtained from laying hens-immunized inactivated whole H1N1 virus reduced the H1N1 viral infection in MDCK cells [40]. Additional *in vivo* analysis has demonstrated that the anti-H1N1 IgY provided protection against the virus by reducing the infectious titer of the virus in the lung of mouse model [40]. To our knowledge, this is the first report demonstrating the level of HI and NT antibodies against influenza A virus which could imply the protective immunity after influenza virus infection in farmed crocodiles. In addition, the use of crocodile IgY can be an alternative approach for diagnostic and therapeutic applications of influenza virus and other infectious diseases in crocodiles.

Although the crocodiles showed no active viral infection at the time of sample collection, we attempted to investigate the susceptibility of avian and human influenza virus in Siamese crocodile host cells. Based on the prior findings of IAV antibodies by HI and NT assays, we chose virus strains that correspond to the virus subtypes found to be positive in both assays for experimental infection in primary Siamese crocodile fibroblast cells. The viral infection experiments clearly showed that all the tested wildtype strains of the influenza viruses (avian H1, H2, H9 and human H1) could infect and replicate within the primary Siamese crocodile fibroblast cells. Our results were similar to a previous study that demonstrated the ability of primary American alligator (*Alligator mississippiensis*) cell line, and embryos for supporting virus replication of four LPAI strains, human H1N1 or HPAI H5N1 viruses [14]. The evidence of virus replication has been determined by the detection of the influenza virus M gene and infectious virus in allantoic fluid as well as the staining of the virus antigen in embryo tissues [14].

Several limitations in our study need to be addressed. Firstly, the sample sizes were small leading to decreased confidence in apparent prevalence result. The prevalence obtained from the small sample size was not suitable to reflect the situation of the virus infection in the entire population of the farmed crocodiles particularly the true prevalence of each sampling year. The difficulties associated with the sample collection from crocodiles could affect the available sample types as well as the sample volume required for testing. Secondly, the reference strains of LPAI viruses and one strain of the human H1 influenza (TH104) virus in the HI and NT assays were not the current circulating isolates. Moreover, because of no report on the HPAI virus circulation among animal reservoirs in Thailand, the reference strains of HPAI viruses were not included in the serological assays of our study. The limitation of the tested viruses may have resulted in underestimating IAV subtype prevalence among the crocodile population. Thirdly, we had limited evidence for IAV carriage in farmed crocodiles. We were unable to detect any viral RNA from the pharyngeal swabs of the crocodiles resulting in lack of genomic information related to the epidemiology of the virus.

## Conclusions

Our findings revealed the evidence of the past infection of avian and human influenza viruses in Siamese crocodiles in central Thailand. The serological investigation suggested that the farmed Siamese crocodiles were susceptible to influenza virus subtypes, including avian H1, H2, H9, and human H1 (TH104). Although influenza viral RNA was not detected in any of the tested pharyngeal swab samples, the further analyses on wildtype virus infection in primary Siamese crocodile fibroblasts confirmed the ability of avian H1, H2, H9 and human H1 influenza viruses to infect and replicate within crocodilian host cells. Our results highlight the role of crocodile species in the ecology of IAV particularly the potential to serve as the reservoir or mixing vessel for the viruses that significantly threaten both human and animal health.

## Supporting information

S1 Table Demographic and HI/NT antibody titers of 43 individual crocodiles with seropositive against subtypes of influenza A virus. (DOCX)

S1 Fig Raw image. Original, uncropped, and unprocessed image supporting Fig 2.
(PDF)
