# Supplementary material for: Evidence of avian and human influenza A virus infection in farmed Siamese crocodiles (Crocodylus siamensis) in Thailand
Source: PLoS One. 2025 Jan 7;20(1):e0317035. doi: 10.1371/journal.pone.0317035 (PMC11706503; doi:10.1371/journal.pone.0317035)
Supplement: S1 Table — (DOCX) [file pone.0317035.s001.docx]

**Table S1.** Demographic and HI/NT antibody titers of 43 individual crocodiles with seropositive against subtypes of influenza A virus.

| **No** | **Date of collection** | **Age** | **Place** | **Province** | **AIV H1** | | **AIV H2** | | **AIV H9** | | **Human H1** | |
| --- | --- | --- | --- | --- | --- | --- | --- | --- | --- | --- | --- | --- |
|  |  |  |  |  | **HI** | **NT** | **HI** | **NT** | **HI** | **NT** | **HI** | **NT** |
| 1 | Jan 12, 2012 | 5 Y | Farm A | Nakhon Pathom | <20 | ND | <20 | ND | 20 | 20 | <20 | ND |
| 2 | Jan 12, 2012 | 5 Y | Farm A | Nakhon Pathom | <20 | ND | <20 | ND | 20 | 20 | <20 | ND |
| 3 | Jan 12, 2012 | 5 Y | Farm A | Nakhon Pathom | <20 | ND | <20 | ND | 20 | 20 | <20 | ND |
| 4 | Jan 12, 2012 | 5 Y | Farm A | Nakhon Pathom | <20 | ND | <20 | ND | 20 | 20 | <20 | ND |
| 5 | Jan 12, 2012 | 5 Y | Farm A | Nakhon Pathom | <20 | ND | <20 | ND | 20 | 20 | <20 | ND |
| 6 | Jan 12, 2012 | 5 Y | Farm A | Nakhon Pathom | <20 | ND | <20 | ND | 40 | 20 | <20 | ND |
| 7 | Jan 12, 2012 | 5 Y | Farm A | Nakhon Pathom | <20 | ND | <20 | ND | 20 | 20 | <20 | ND |
| 8 | Jan 12, 2012 | 5 Y | Farm A | Nakhon Pathom | <20 | ND | <20 | ND | 40 | 40 | <20 | ND |
| 9 | Jan 12, 2012 | 5 Y | Farm A | Nakhon Pathom | <20 | ND | <20 | ND | 20 | 20 | <20 | ND |
| 10 | Jan 12, 2012 | 5 Y | Farm A | Nakhon Pathom | <20 | ND | <20 | ND | <20 | ND | 80 | 80 |
| 11 | Jan 12, 2012 | 5 Y | Farm A | Nakhon Pathom | <20 | ND | <20 | ND | <20 | ND | 20 | 80 |
| 12 | Jan 12, 2012 | 5 Y | Farm A | Nakhon Pathom | <20 | ND | <20 | ND | <20 | ND | 20 | 40 |
| 13 | Jan 12, 2012 | 5 Y | Farm A | Nakhon Pathom | <20 | ND | <20 | ND | <20 | ND | 40 | 40 |
| 14 | Jan 12, 2012 | 5 Y | Farm A | Nakhon Pathom | <20 | ND | <20 | ND | <20 | ND | 20 | 40 |
| 15 | Jan 12, 2012 | 5 Y | Farm A | Nakhon Pathom | <20 | ND | <20 | ND | <20 | ND | 20 | 40 |
| 16 | Aug 2, 2018 | 1Y | Farm B | Nakhon Pathom | <20 | ND | <20 | ND | 20 | 40 | <20 | ND |
| 17 | Aug 2, 2018 | 1Y | Farm B | Nakhon Pathom | <20 | ND | <20 | ND | 40 | 20 | <20 | ND |
| 18 | Aug 2, 2018 | 1Y | Farm B | Nakhon Pathom | <20 | ND | <20 | ND | 20 | 20 | <20 | ND |
| 19 | Aug 2, 2018 | 1Y | Farm B | Nakhon Pathom | <20 | ND | <20 | ND | 80 | 40 | <20 | ND |
| 20 | Aug 2, 2018 | 1Y | Farm B | Nakhon Pathom | <20 | ND | <20 | ND | 20 | 20 | <20 | ND |
| 21 | Aug 2, 2018 | 1Y | Farm B | Nakhon Pathom | <20 | ND | <20 | ND | 80 | 40 | <20 | ND |
| 22 | Aug 2, 2018 | 1Y | Farm B | Nakhon Pathom | <20 | ND | <20 | ND | 20 | 40 | <20 | ND |
| 23 | Aug 2, 2018 | 1Y | Farm B | Nakhon Pathom | <20 | ND | <20 | ND | 20 | 40 | <20 | ND |
| 24 | Jul 30, 2019 | 1Y | Farm E | Sing Buri | <20 | ND | 20 | 40 | <20 | ND | <20 | ND |
| 25 | Jul 30, 2019 | 1Y | Farm E | Sing Buri | <20 | ND | 20 | 40 | <20 | ND | <20 | ND |
| 26 | Jul 30, 2019 | 1Y | Farm E | Sing Buri | <20 | ND | 20 | 40 | <20 | ND | <20 | ND |
| 27* | Jul 30, 2019 | 1Y | Farm E | Sing Buri | 20 | 20 | 40 | 40 | <20 | ND | <20 | ND |
| 28 | Jul 30, 2019 | 1Y | Farm E | Sing Buri | <20 | ND | 20 | 40 | <20 | ND | <20 | ND |
| 29 | Jul 31, 2019 | 7 mo | Farm G | Suphan Buri | <20 | ND | 40 | 40 | <20 | ND | <20 | ND |
| 30 | Jul 31, 2019 | 7 mo | Farm G | Suphan Buri | <20 | ND | 20 | 40 | <20 | ND | <20 | ND |
| 31 | Sep 4, 2019 | 4 mo | Farm H | Saraburi | <20 | ND | 20 | 20 | <20 | ND | <20 | ND |
| 32 | Sep 4, 2019 | 4 mo | Farm H | Saraburi | <20 | ND | 20 | 40 | <20 | ND | <20 | ND |
| 33* | Sep 4, 2019 | 4 mo | Farm H | Saraburi | 20 | 20 | 20 | 40 | <20 | ND | <20 | ND |
| 34* | Sep 4, 2019 | 4 mo | Farm H | Saraburi | 20 | 20 | 40 | 80 | <20 | ND | <20 | ND |
| 35 | Sep 4, 2019 | 4 mo | Farm H | Saraburi | <20 | ND | 40 | 40 | <20 | ND | <20 | ND |
| 36 | Sep 4, 2019 | 4 mo | Farm H | Saraburi | <20 | ND | 20 | 80 | <20 | ND | <20 | ND |
| 37 | Sep 17, 2019 | 1 Y | Farm J | Lop Buri | <20 | ND | 20 | 20 | <20 | ND | <20 | ND |
| 38 | Sep 30, 2019 | 1 Y | Farm M | Chai Nat | <20 | ND | 40 | 20 | <20 | ND | <20 | ND |
| 39 | Sep 30, 2019 | 1 Y | Farm M | Chai Nat | <20 | ND | 20 | 20 | <20 | ND | <20 | ND |
| 40 | Nov 11, 2019 | 7 mo | Farm N | Ayutthaya | <20 | ND | 20 | 20 | <20 | ND | <20 | ND |
| 41 | Nov 11, 2019 | 7 mo | Farm N | Ayutthaya | <20 | ND | 20 | 20 | <20 | ND | <20 | ND |
| 42 | Nov 11, 2019 | 7 mo | Farm N | Ayutthaya | <20 | ND | 20 | 40 | <20 | ND | <20 | ND |
| 43 | Nov 11, 2019 | 7 mo | Farm N | Ayutthaya | <20 | ND | 20 | 80 | <20 | ND | <20 | ND |
|  |  |  |  | Total | 3 | | 20 | | 17 | | 6 | |

ND = not determined, Y = Year, mo = month, HI = hemagglutination inhibition antibody titer, NT = neutralizing antibody titer,
AIV = avian influenza virus, Human = human influenza virus.

*: Individual crocodile gave seropositive results for both virus subtypes.

Both HI and NT titer of ≥20 were considered as seropositive. Only HI titer ≥20 was further determined for NT titer.
