# Supplementary material for: Evidence of avian and human influenza A virus infection in farmed Siamese crocodiles (Crocodylus siamensis) in Thailand
Source: PLoS One. 2025 Jan 7;20(1):e0317035. doi: 10.1371/journal.pone.0317035 (PMC11706503; doi:10.1371/journal.pone.0317035)
Supplement: S1 Fig — Original, uncropped, and unprocessed image supporting Fig 2. (PDF) [file pone.0317035.s002.pdf]

RAW DATA OF WESTERN BLOT ANALYSIS

GEL 2A

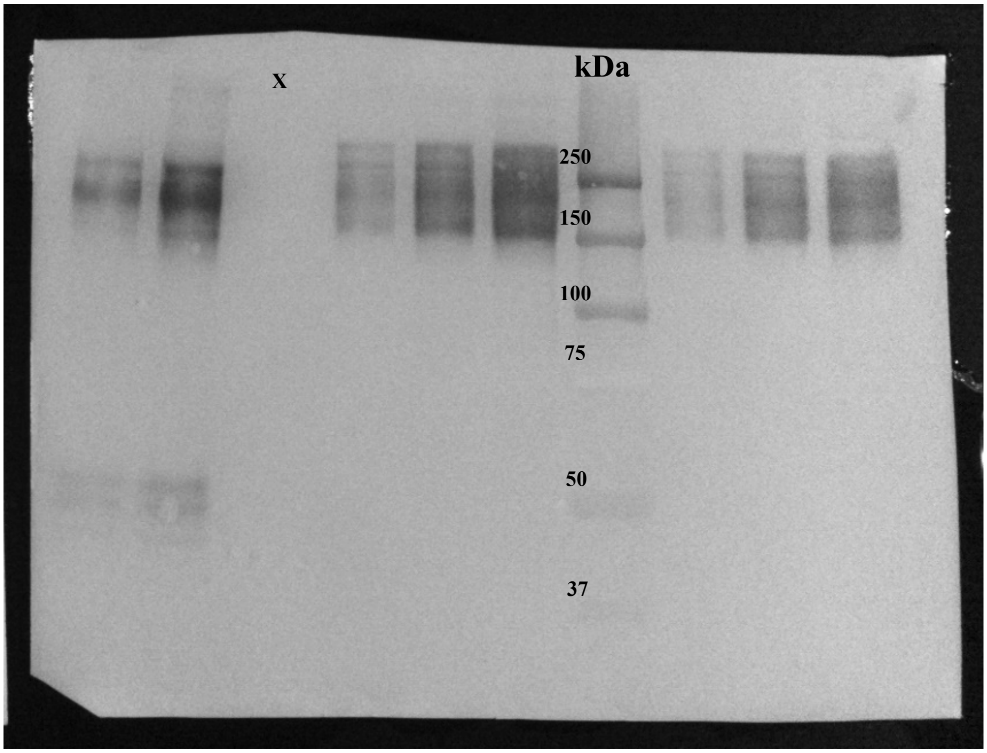

1. Chicken serum (1:100)
2. Chicken serum (1:50)
3. -----
4. Plasma no.1 (1:200)
5. Plasma no.1 (1:100)
6. Plasma no.1 (1:50)
7. Precision Plus Protein™ Standards (Bio-Rad Laboratories)
8. Plasma no.2 (1:200)
9. Plasma no.2 (1:100)
10. Plasma no.2 (1:50)

GEL 2B

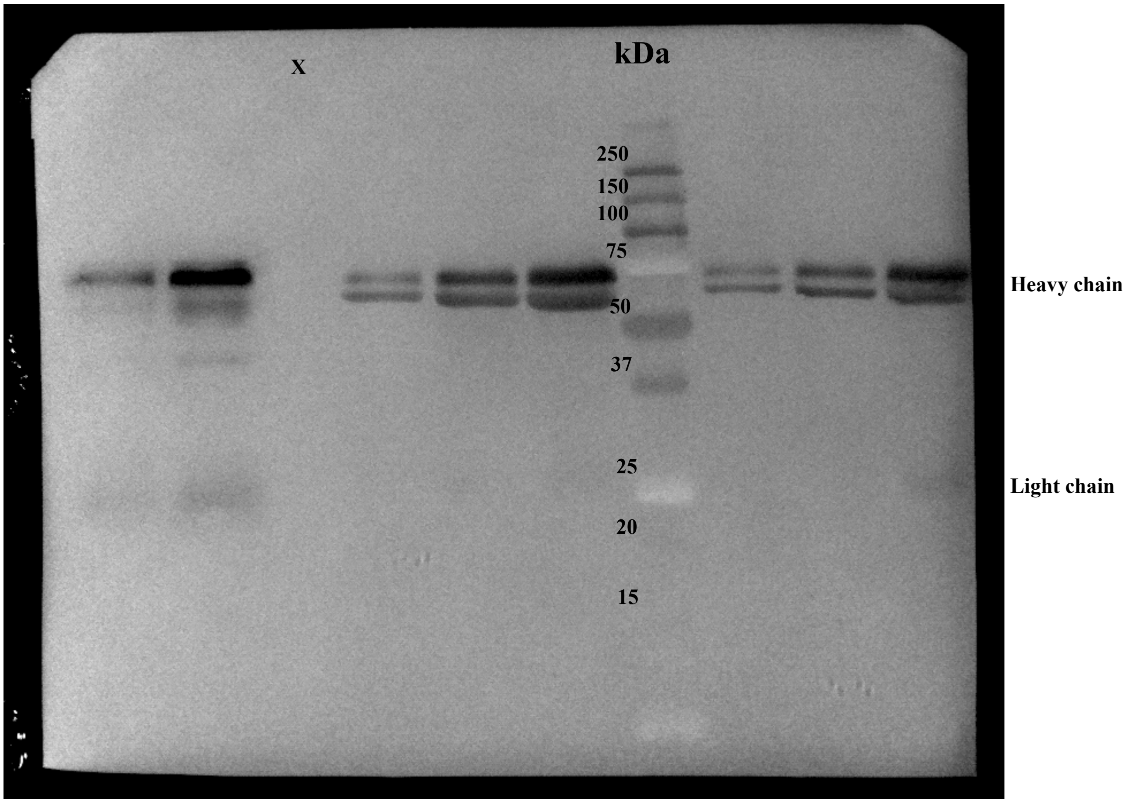

1. Chicken serum (1:100)
2. Chicken serum (1:50)
3. -----
4. Plasma no.1 (1:200)
5. Plasma no.1 (1:100)
6. Plasma no.1 (1:50)
7. Precision Plus Protein™ Standards (Bio-Rad Laboratories)
8. Plasma no.2 (1:200)
9. Plasma no.2 (1:100)
10. Plasma no.2 (1:50)
